# Prediction of effective humoral response to SARS-CoV-2 vaccines in healthy subjects by cortical thickness of post-vaccination reactive lymphadenopathy

**DOI:** 10.1007/s00330-023-09662-5

**Published:** 2023-05-05

**Authors:** Alba Cristina Igual-Rouilleault, Ignacio Soriano, Paola Leonor Quan, Gabriel Reina, José Luis del Pozo, Álvaro Gónzalez, Leire Fernández-Ciriza, Alejandro Fernández-Montero, Luis Pina, Arlette Elizalde

**Affiliations:** 1 Department of Radiology, Clínica Universidad de Navarra, Avenida Pío XII 36, Pamplona, Spain; 2 Department of Allergy and Clinical Immunology, Clínica Universidad de Navarra, Avenida Pío XII 36, Pamplona, Spain; 3 Clinical Microbiology Unit, Clínica Universidad de Navarra, Avenida Pío XII 36, Pamplona, Spain; 4 Infectious Diseases Division, Clínica Universidad de Navarra, Avenida Pío XII 36, Pamplona, Spain; 5 Department of Clinical Microbiology, Clínica Universidad de Navarra, Avenida Pío XII 36, Pamplona, Spain; 6 Laboratory of Biochemistry, Clínica Universidad de Navarra, Avenida Pío XII 36, Pamplona, Spain; 7 Clinical Microbiology Unit, Clínica Universidad de Navarra, Avenida Pío XII, 36 Pamplona, Spain; 8 Prevention and Health Service, Clínica Universidad de Navarra, Avenida Pío XII, 36 Pamplona, Spain; 9 Breast Imaging Unit, Department of Radiology, Clínica Universidad de Navarra, Avenida Pío XII, 36 Pamplona, Spain

**Keywords:** COVID-19 vaccines, Lymphadenopathy, Healthy volunteers

## Abstract

**Purpose:**

To study the association between ultrasound cortical thickness in reactive post-vaccination lymph nodes and the elicited humoral response and to evaluate the performance of cortical thickness as a predictor of vaccine effectiveness in patients with and without a previous history of COVID-19 infection.

**Methods:**

A total of 156 healthy volunteers were recruited and followed prospectively after receiving two COVID-19 vaccination doses using different protocols. Within a week after receiving the second dose, an axillary ultrasound of the ipsilateral vaccinated arm was performed, and serial post-vaccination serologic tests (PVST) were collected. Maximum cortical thickness was chosen as a nodal feature to analyze association with humoral immunity. Total antibodies quantified during consecutive PVST in previously-infected patients and in coronavirus-naïve volunteers were compared (Mann–Whitney *U* test). The association between hyperplastic-reactive lymph nodes and effective humoral response was studied (odds ratio). The performance of cortical thickness in detecting vaccination effectiveness was evaluated (area under the ROC curve).

**Results:**

Significantly higher values for total antibodies were observed in volunteers with a previous history of COVID-19 infection (*p *< 0.001). The odds ratio associating immunized coronavirus-naïve volunteers after 90 and 180 days of the second dose with a cortical thickness ≥ 3 mm was statistically significant (95% CI 1.52–6.97 and 95% CI 1.47–7.29, respectively). The best AUC result was obtained comparing antibody secretion of coronavirus-naïve volunteers at 180 days (0.738).

**Conclusions:**

Ultrasound cortical thickness of reactive lymph nodes in coronavirus-naïve patients may reflect antibody production and a long-term effective humoral response elicited by vaccination.

**Clinical relevance statement:**

In coronavirus-naïve patients, ultrasound cortical thickness of post-vaccination reactive lymphadenopathy shows a positive association with protective antibody titers against SARS-CoV-2, especially in the long term, providing new insights into previous publications.

**Key Points:**

• *Hyperplastic lymphadenopathy was frequently observed after COVID-19 vaccination.*

• *Ultrasound cortical thickness of reactive post-vaccine lymph nodes may reflect a long-term effective humoral response in coronavirus-naïve patients.*

## Introduction

Coronavirus disease-19 (COVID-19) is an ongoing global pandemic caused by the 2019 novel coronavirus (2019-nCoV), also known as severe acute respiratory syndrome coronavirus 2 (SARS-CoV-2). It was first detected in the Chinese city of Wuhan in early December 2019, after the notification of numerous cases of atypical pneumonia. Only 1 month later, COVID-19 spread quickly worldwide, and the World Health Organization (WHO) declared it a public health emergency of international concern (PHEIC). On March 2020, it was declared a global pandemic. In consequence, multiple public health interventions were implemented worldwide to decrease the transmission of the virus and contain its spread.

After more than 2 years of the COVID-19 outbreak, the only measure guaranteeing virus control has been the implementation of broad vaccination programs. Different types of vaccines have obtained emergency use authorizations (EUA) from the US Food and Drug Administration (FDA) and the European Medical Agency (EMA), including novel mRNA (Pfizer-BioNTech, Moderna) and viral vector-based (AstraZeneca, Janssen) vaccines. Additionally, in June 2021, the WHO's Strategic Advisory Group of Experts on Vaccines approved “mix-and-match” or heterologous COVID-19 vaccine protocols with an initial dose of AstraZeneca and a booster of Pfizer vaccine.

All COVID-19 vaccine platforms are administered intramuscularly but their mechanism of action differs: mRNA vaccines use the virus’s genetic material (RNA) during inoculation while viral vectors used modified versions of a different virus [1]. Their subsequent immune response is a complicated process necessitating several steps, many of which involve the lymphatic system [2]. Vaccine antigens are taken up by antigen-presenting cells (APCs) from the injection site to regional secondary lymphoid tissues, namely the lymph nodes, via lymphatic channels. Lymph nodes represent the activation center of the immune response, where vaccine-peptide antigens are presented by APCs to residing lymphocytes in order to elicit two main responses: cellular response, with the formation of cytotoxic T lymphocytes capable of directly killing infected cells, and a humoral response, which depends on B cells proliferation in the lymph node’s germinal center (GC), resulting in the formation of matured memory B-cells and antibody-secreting long-lived plasma cells [3].

Therefore, widespread cases of reactive unilateral axillary lymphadenopathy were reported after the introduction of the COVID-19 mass vaccination campaign [4–12]. While axillary and supraclavicular adenopathy ipsilateral to the injection site has also been documented after administration of other vaccines in the past [13–17], no data exist on the correlation between vaccination-associated reactive lymphadenopathy and the elicited immune response to anti-SARS-CoV-2 vaccines. The aim of this study is to assess, in healthy adults, a potential association between the phenomenon of reactive hyperplastic lymphadenopathy identified on post-vaccination ultrasound and the humoral immunity revealed by serologic testing.

## Material and methods

### Study design

Between February and December 2021, 247 employees from our center, with no previous history of cancer, were recruited in this prospective observational single-center study. All volunteers gave written informed consent and the Ethical Committee of Clinical Research from our center approved the study protocol. Patients were included after receiving two doses of COVID-19 vaccination with either Pfizer, Moderna, AstraZeneca, or a mix-and-match COVID-19 vaccine protocol. The administration of both doses was performed in the same arm (preferably in the non-dominant arm). Of these 247 participants, 156 underwent serologic testing following the second vaccine dose with a median time interval between the booster vaccine dose and serology testing of 21, 90, and 180 days. The vaccines administered, demographic data (age and sex), and prior history of COVID-19 infection, were recorded for all of them.

### Ultrasound acquisition and data assessment

Ultrasounds were obtained using two different broad-band linear transducers with a band frequency of 8-13 MHz (Logic E9, GE Healthcare, and Aplio i800 series ultrasound system, Canon Medical Systems Corporation). All volunteers underwent an axillary ultrasound (US) of the ipsilateral vaccinated arm within a week after the second dose. Ultrasound scans were performed by two third-year residents and two radiologists with more than 20 years of experience in breast imaging. Prior to participating in the study, the two third-year residents who took part in the research had completed their breast imaging rotation. Additionally, they had conducted an observer training of axillary ultrasound performance with the two expert radiologists in order to perform US scans independently.

**Fig. 1:**
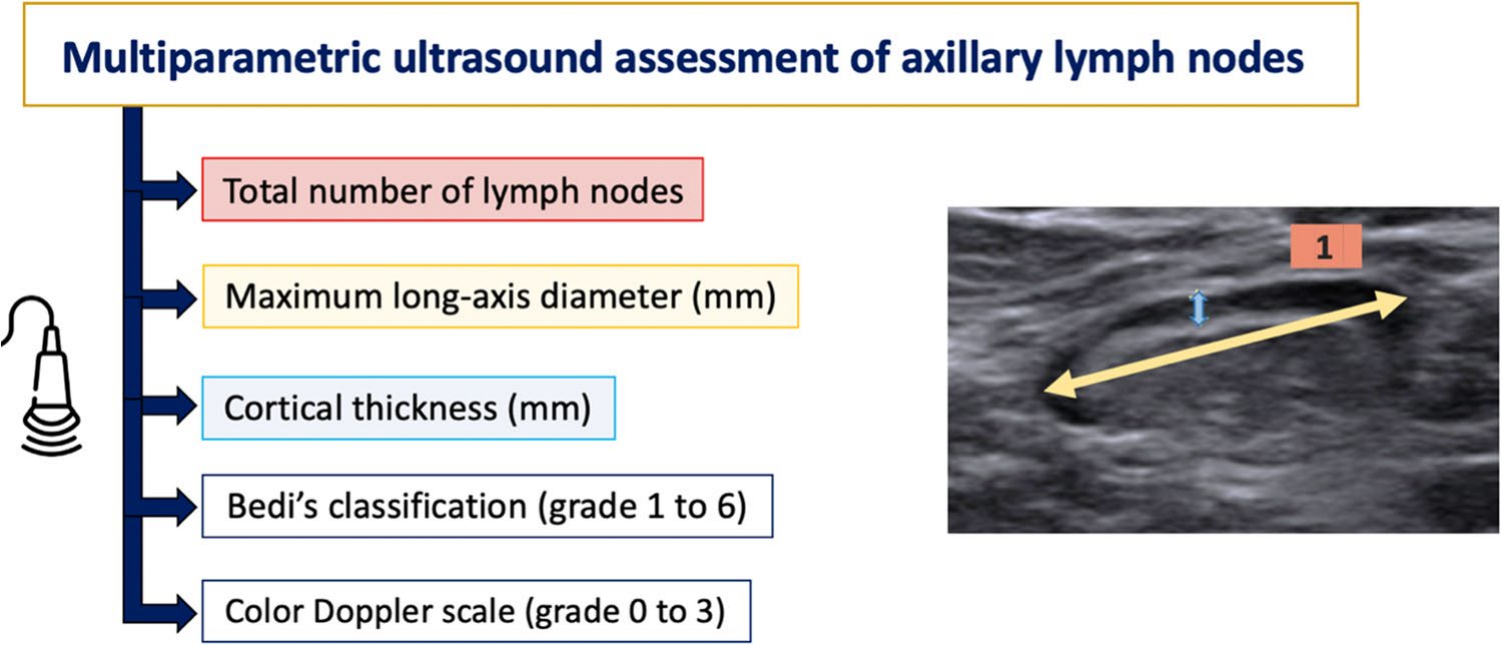
Enumeration of the five nodal imaging features assessed on ultrasound (total number of visible nodes, maximum measurement of long-axis size, maximum measurement of cortical thickness, morphological Bedi’s classification, and color Doppler evaluation). US image illustrating how the three objective parameters recorded in the study were performed

All axillary levels ipsilateral to the vaccine injection were evaluated and the following nodal imaging features were assessed: total number of visible lymph nodes, maximum measurements of long-axis size and cortical thickness, morphological Bedi’s classification, and color Doppler evaluation (Fig. 1). However, the variable used to analyze the association between morphologic changes in regional lymph nodes and antibody levels was only the maximum cortical thickness. All patients’ images, regardless of whether the examinations were performed by the two expert radiologists (67% of US scans) or by the two third-year residents (33% of US scans), were reviewed by the two radiologists with more than 20 years of experience in breast imaging, to reduce inter-observer variability and to select, in consensus, the appropriate cortical thickness measurement for each patient.

### Humoral Response Evaluation

Anti-SARS-CoV-2 antibody detection was performed using two different commercial chemoluminescence tests. On the one hand, quantification of total antibodies (IgG+IgM) against the receptor binding domain (RBD) of SARS-CoV-2 spike (S) protein was performed using Elecsys® Anti-SARS-CoV-2 S (Roche Diagnostics) test in the cobas e601 platform. On the other hand, the detection of total antibodies (IgG+IgM) against viral nucleocapsid (Anti-N) was performed using Elecsys® Anti-SARS-CoV-2 test (Roche Diagnostics).

The package insert of Anti-S-RBD testing indicated that a quantification above 0.8 U/ml must be considered positive or reactive. Regarding Anti-S-RBD levels we used a threshold of 1000 U/mL. An Anti-S-RBD quantification below 1000 U/mL, was considered a low (or non-protective) antibody level, and an Anti-S-RBD quantification of 1000 U/mL or more was considered a high (protective) antibody level. This interpretation was based on previous studies describing a waning in humoral immunity if levels detected for anti-S IgG were 1000 U/mL or less [18–20].

The package insert of Anti-N antibodies recommends a positive interpretation if the results are above 1.0 COI. However, according to previously reported studies [21], we selected 0.150 COI as a cut-off to improve the marker’s sensitivity.

### Statistical analysis

Data collection was recorded in an EXCEL database (Microsoft). All statistical analyses were performed using SPSS Statistics Version 21 (IBM) and for all comparisons, a *p* value < 0.05 was considered statistically significant.

Categorical variables were reported as frequency and percentage. Continuous variables were evaluated for normal distribution and reported as the median and interquartile range (IQR). Assessment of gender, age, and vaccine protocol administered between coronavirus-naïve volunteers and previously infected patients was performed using Fisher’s exact test, Mann–Whitney *U* test, and chi-square test, respectively. Additionally, a Mann–Whitney *U* test was also applied to compare serial antibody titers obtained 21, 90, and 180 days from the second vaccine dose between both groups. An odds ratio test was performed to investigate the association between hyperplastic cortical thickness (≥ 3 mm) and effective humoral immune response (IgG+IgM ≥ 1000 U/mL) with the same subgroup analyses.

Finally, the area under the ROC curve (AUC) was calculated to evaluate the performance of the cortical thickness to detect the *effectiveness* of *COVID*-19 *vaccines* over time. Areas under the curve (AUC) were constructed by calculating the sensitivities (true positive rate) and specificities (false positive rate) of the cortical thickness at several cut-off points considering antibody levels ≥ 1000 U/mL as protective. “The best cut-off” points were selected taking two elements into account: a cortical thickness slightly above 3 mm and a discreetly higher sensitivity to specificity. Our objective was to study the presence of high (protective) Anti-S IgG levels. Therefore, we prioritized the sensitivity of the algorithm while preserving adequate specificity.

## Results

### Patient characteristics

Out of 247 included volunteers who fulfilled the inclusion criteria, serial serological tests were performed for 156 individuals. Demographic data analysis (*n*=156) showed an average age of 44.01 ± 11.56 (range 20–65 years) and a large percentage of women (91.7%). According to the COVID-19 vaccine protocols administered, subjects were divided into four groups: recipients of the Pfizer (45; 28.8%), Moderna (28; 17.9%), AstraZeneca (60; 38.5%) vaccines or mix-and-match COVID vaccination (23; 14.7%). No significant differences were seen when comparing the gender and the mean age of patients between coronavirus-naïve volunteers and previously infected patients. However, comparative analyses of vaccine protocols between coronavirus-naïve volunteers and previously infected patients showed a statistically significant difference because almost all the convalescent volunteers were vaccinated with the Pfizer vaccine (Table 1). Total antibody levels of recruited patients are summarized in Table 2, which also includes analysis by subgroups.

**Table 1:** Details of sample. Categorical variables are reported as frequency and percentage; Continuous variables are reported as mean ± standard deviation (SD)

	Total number of recipients with serology testing (*n* = 156)	Coronavirus-naïve volunteers (*n* = 137)	Convalescent volunteers (*n* = 19)	*p* value
**Gender** (men/women)	13/143 (8.3%/91.7%)	10/127 (11.2%/ 88.8%)	3/16 (15.8%/84.2%)	**0.198**
**Age y/o** (mean ± SD)	44.01 ± 11.56	43.96 ± 11.41	44.32 ± 12.92	**0.871**
**Vaccine protocol**				***< 0.001***
Pfizer-Pfizer	45 (28.8%)	28 (20.44%)	17 (89.47%)	
Moderna-Moderna	28 (17.9%)	28 (20.44%)	0	
AstraZeneca-AstraZeneca	60 (38.5%)	58 (42.34%)	2 (10.53%)	
AstraZeneca-Pfizer	23 (14.7%)	23 (16.78%)	0	

Values highlighted in bold are statistically significant (value inferior to 0.001)

**Table 2:** Comparison of median ± interquartile range (IQR) values for total antibody levels collected 21, 90, and 180 days from second booster dose between the three different vaccine protocols

	Total number of recipients with serology testing* (n* = 156)	Coronavirus-naïve volunteers (*n* = 137)	Convalescent volunteers (*n* = 19)	*p* value
**Total antibodies 21 days from Vac-2**	**3546 ± 6608**	**3189 ± 4129**	**17933 ± 18055**	**<0.001**
mRNA (Pfizer-BioNTech, Moderna)	4630 ± 6474.5	3595.5 ± 3446	20334 ± 16484.5	
Viral vector-based (AstraZeneca-AstraZeneca)	2402.5 ± 1834	2377 ± 1820.5	3178 ± 736	
Mix-and-match (AstraZeneca-Pfizer)	16340 ± 13614	16340 ± 13614	-	
**Total antibodies 90 days from Vac-2**	**1831 ± 2992.7**	**1413 ± 2210**	**8228 ± 8370**	**< 0.001**
mRNA (Pfizer-BioNTech, Moderna)	2520 ± 3217	2263.5 ± 1793	9230 ± 18232	
Viral vector-based (AstraZeneca-AstraZeneca)	790.1 ± 776.5	785.6 ± 727.8	± 43	
Mix-and-match (AstraZeneca-Pfizer)	3762 ± 3546	3762 ± 3546	-	
**Total antibodies 180 days from Vac-2**	**1006 ± 1368.8**	**899.5 ± 1172.1**	**3342.5 ± 3766.3**	**<0.001**
mRNA (Pfizer-BioNTech, Moderna)	1403 ± 1624	1163 ± 747.7	3900 ± 3848.5	
Viral vector-based (AstraZeneca-AstraZeneca)	389.3 ± 418.3	378.4 ± 431.1	656.7	
Mix-and-match (AstraZeneca-Pfizer)	1524 ± 1229.6	1524 ± 1229.6	-	

Values highlighted in bold are statistically significant (value inferior to 0.001)

### Vaccine-associated cortical nodal thickness and post-vaccination antibody secretion

To study the association between the cortical thickness of axillary lymph nodes (exposure) and the humoral response quantified by PVST (outcome), we defined lymph nodes showing a *cortical thickness* greater than 3 mm *as morphologically* reactive lymphadenopathy, and a total antibody (IgG+IgM) quantification equal or greater than 1000 U/mL as a protective serologic level. The odds of effective humoral response could not be calculated in the subgroup of previously infected patients because of the small sample. However, in coronavirus naïve volunteers, we found statistically significant odds of effective humoral response at 90 days (3.25, 95% CI 1.52–6.97) and at 180 days (3.28, 95% CI 1.47–7.29).

### Receiver operating characteristic curves analysis

**Fig. 2:**
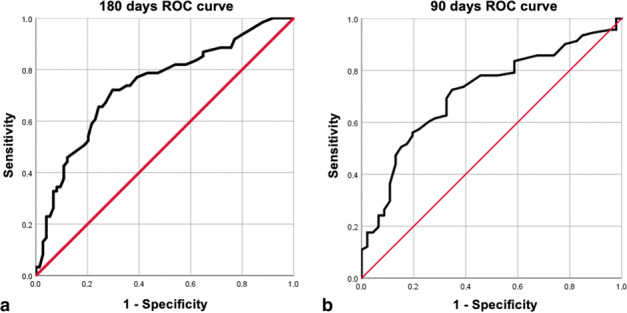
**a** ROC curve for coronavirus-naïve volunteers at 180 days with a cortical thickness of 3.55 mm as the best cut-off point with a sensitivity of 73.8% and a specificity of 63.5%. **b** ROC curve for coronavirus-naïve volunteers at 90 days with a cortical thickness cut-off of 3.55 with a sensitivity of 72.5% and a specificity of 65.2%.

Areas under the curve (AUC) in convalescent volunteers suggest that cortical thickness holds no diagnostic ability in this subgroup (AUC < 0.50). Regarding the coronavirus-naïve volunteers’ subgroup, the higher AUC value was obtained comparing antibody secretion of coronavirus-naïve volunteers at 180 days (AUC=0.738) with a cortical thickness of 3.55 mm as the best cut-off point with a sensitivity of 73.8% and a specificity of 63.5%. This was followed by antibody secretion of coronavirus-naïve volunteers at 90 days (AUC = 0.713) with a cortical thickness of 3.35 mm as the best cut-off point with a sensitivity of 72.5% and a specificity of 65.2% (Fig. 2); and finally, by antibody secretion of coronavirus-naïve volunteers at 21 days (AUC = 0.670) with a cortical thickness of 3.35 mm as the best cut-off point with a sensitivity of 63.3% and a specificity of 64.7% (AUC = 0.670).

## Discussion

Ever since the rollout of vaccination programs to the entire population, widespread cases of hyperplastic lymph nodes have been reported as ipsilateral to the injection site. However, the association between lymphadenopathy induced by COVID-19 vaccinations and immune response elicited remains unknown. This is the first prospective study that examined the association between cortical morphologic features of reactive axillary lymph nodes and SARS-CoV-2 antibody levels in healthy patients, adding new insights into this clinical pandemic-era conundrum.

All patients recruited in our prospective cohort were employees from our center and, after requesting their written consent, we were allowed to access their personal medical history. No rheumatological or oncological diseases were present at the time of their inclusion in the study or during their follow-up period, and they were therefore considered healthy volunteers. In the study, women represented the largest percentage of the sample (92%) due to a higher proportion of female employees in the health care and social sector [22]. The mean age of recruited patients was similar to the mean age of the population in our country (44 years old) [23].

**Fig. 3:**
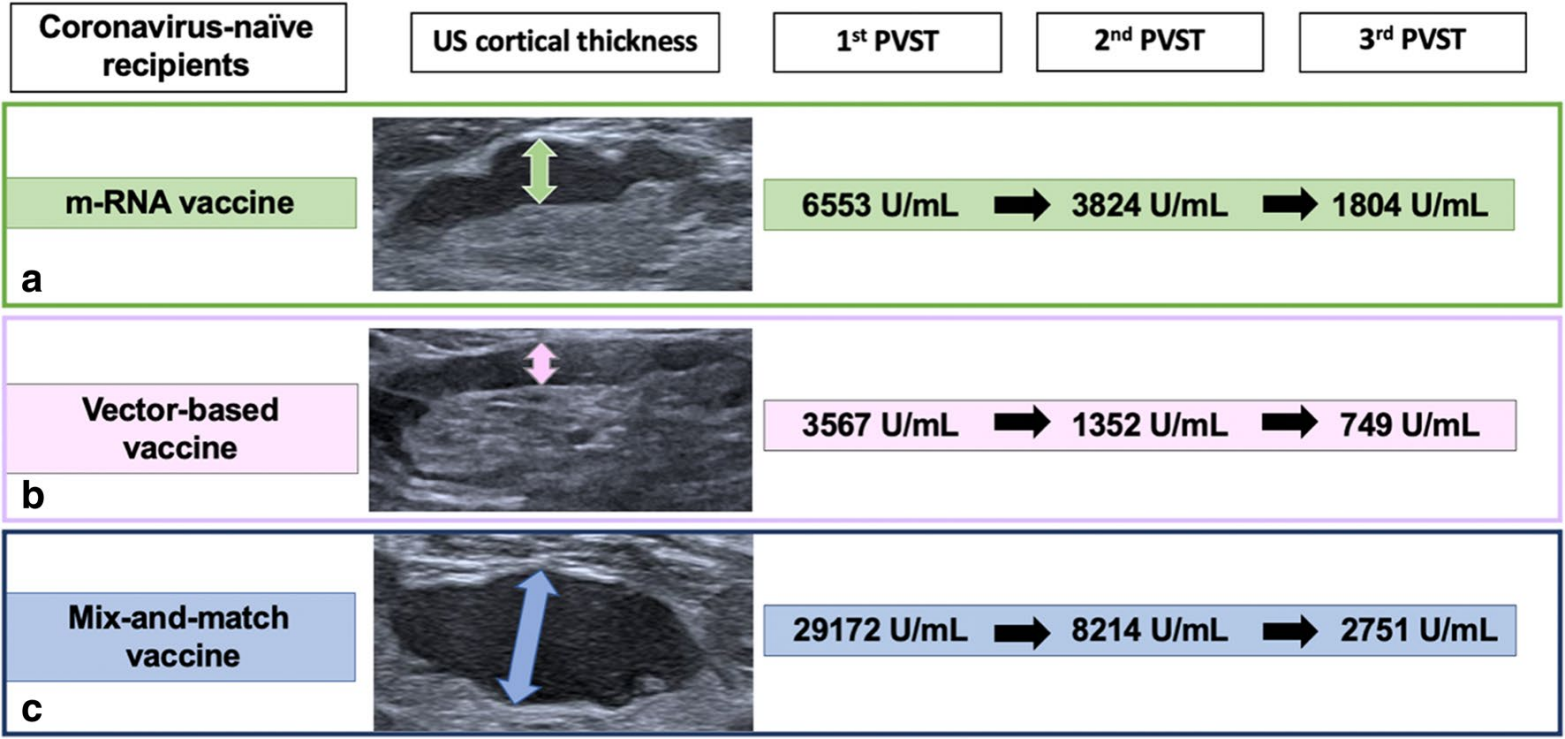
Comparative US images and sequential PSVT results from examples of vaccine recipients with different vaccination protocols. **a** A maximum cortical thickness of 4.5 mm in a type Bedi 4 node seen in a volunteer vaccinated with Moderna m-RNA protocol. **b** A maximum cortical thickness of 3 mm in a type Bedi 3 node detected in a volunteer vaccinated with viral vector-based protocol. **c** A large maximum cortical thickness of 10 mm with absent hilum in a type Bedi 6 node seen in a volunteer after receiving the mix-and-match protocol

Ultrasound examination was obtained from the axillary region ipsilateral to the vaccine injection, including the three axillary levels. In our experience, the most frequently hyperplastic reported lymph nodes were located below the lower edge of the pectoralis minor muscle (level I), so this region was more carefully evaluated. The five ultrasound nodal characteristics described in the design of the study were assessed for each visible node in all volunteers. However, the nodal feature chosen to establish an association with antibody levels was the maximum value detected of cortical thickness. This variable is the most relevant clinical-radiological factor, and it is a quantitative measurement with more objectivity and reproducibility as well as an easier statistical assessment. So, regardless of whether a lymph node presented a diffuse and symmetric cortical thickness greater than or equal to 3 mm (type Bedi 3), a generalized lobulated cortical thickening (type Bedi 4), or a focal cortical lobulation (type Bedi 5), we chose its maximum cortical value (measured in millimeters) (Fig. 3).

Before studying the association between the maximum cortical thickness of reactive lymphadenopathy and the quantification of total antibodies, we avoided the possible effect of a previous history of COVID-19 disease in the humoral immune response by dividing the sample into two subgroups: coronavirus-naïve volunteers and previously infected patients. We had two strategies to detect incident cases of COVID-19 among our participants: one, based on information from their clinical record when results of PCR or antigen detection were available and, the second one, based on serological follow-up every 1–3 months. The sequential post-vaccination serologic tests (PVST) performed included the detection of antibodies against both the spike (Anti-S) and the nucleocapsid (Anti-N). All COVID-19 vaccine platforms induce spike protein-specific neutralizing antibodies (Anti-S). However, antibodies against the nucleocapsid (Anti-N) are only generated by natural infection, so their detection in patients (using a cut-off of 0.150) allowed us to diagnose additional COVID-19 cases not detected by direct testing.

Data analysis confirmed statistically higher SARS-CoV-2 IgG antibody levels in individuals previously diagnosed with COVID-19, regardless of the vaccine type used. When comparing the immune response between different vaccine protocols in both groups, we detected higher levels of antibody levels in patients vaccinated with the mix-and-match protocol. This is true for measurements conducted at 21 days, however, differences between the mRNA protocol and the mix-and-match protocol seem less dramatic at 90 days and at 180 days.

To evaluate the association between post-vaccine lymphadenopathy and the effectiveness of the COVID-19 vaccine over time, we defined lymph nodes with a cortical thickness greater than 3 mm as morphological reactive because, following Bedi’s classification, this value supposes the change between normal lymph node appearance (Bedi’s grade 1 and 2) to a suspicious/reactive lymph node appearance (Bedi’s grade 3, 4, 5 and 6). Normally, this classification is used as a way to objectively classify the appearance of lymph nodes in oncologic staging and the likelihood of malignancy increases with each morphologic type [24]. However, due to the lack of malignant risk factors in our cohort, axillary lymph nodes showing large cortical thickness were regarded as physiologic, reactive post-vaccine lymph nodes.

It was possible to calculate the odds of effective humoral response in the subgroup of coronavirus naïve volunteers and statistically significant odds of effective humoral response were found at 90 and 180 days. The fact that the germinal-center (GC) is located in the nodal cortex [25] could explain why cortical thickness developed in post-vaccine lymphadenitis may be the morphological evidence that suggests a more robust B cell germinal-center (GC) response elicited by COVID-19 vaccines. This feature may also indicate a more effective humoral response and a higher likelihood of antibody production, especially at 90 and 180 days.

Areas under the curve (AUC) in convalescent volunteers suggest that cortical thickness holds no diagnostic capacity in this subgroup. This result could be explained by previously published results, which showed a less significant axillary lymph node response to vaccination in patients who were previously infected by SARS-CoV-2 [26]. Regarding the subgroup of coronavirus-naïve patients, the higher AUC value was obtained by analyzing antibody secretion of coronavirus-naïve volunteers at 180 days with acceptable discrimination (AUC = 0.738), followed by antibody secretion of coronavirus-naïve volunteers at 90 days (AUC = 0.713). In both analyses, the best cut-off point was determined to be at a cortical thickness greater than 3 mm, in line with our previous explanations of cortical thickness associated with reactive post-vaccine lymphadenopathy. According to these results, we can conclude that the presence of draining lymph nodes with cortical thickness greater than 3 mm is useful in detecting the effectiveness of the COVID-19 vaccine over time, especially, at 180 days.

The findings in this report are subject to some limitations. Firstly, the immunity to SARS-CoV-2 is likely both humoral and cellular, and this second aspect of response one was not analyzed. Secondly, the selection of healthy employees from our center as the target population eliminated the possibility to report nodal feature changes in extreme ages (sample selection bias) and in individuals with immunocompromising conditions. Thirdly, the small number of volunteers receiving each vaccine protocol does not allow investigating by subclasses. Furthermore, we did not study other nodal locations. Finally, the absence of a baseline axillary ultrasound of vaccine recipients prior to vaccination eliminated the possibility in this study of reporting an induced cortical thickness from a baseline. On the other hand, the scientific rigor of these results is enhanced by its prospective design and the participants’ very high adherence to serial serologic testing.

In conclusion, the key findings of our study include (1) the positive association between the cortical thickness of draining lymph nodes after vaccination and protective antibody titers against SARS-CoV-2 in coronavirus-naïve patients (especially in the long term), and (2) an adequate discrimination capacity of a cortical thickness greater than 3 mm in predicting post-vaccination effective humoral response. These findings provide new insights into previous publications on the relationship between ultrasound features of post-vaccination lymphadenopathy in the context of COVID-19 and inspire further investigations.

## Abbreviations

2019-nCOV: New coronavirus disease
APCs: Antigen-presenting cells
AUC: Area under a receiver operating characteristic (ROC) Curve
COVID-19: Coronavirus disease 2019
FDA: Food and Drug Administration
mRNA: Messenger ribonucleic acid
PVST: Post-vaccination serologic testing
SARS-CoV2: Severe acute respiratory syndrome coronavirus
US: Ultrasound

## Declarations

### Guarantor

The scientific guarantors of this publication are Dr Pina and Dr Elizalde.

### Statistics and biometry

One of the authors has significant statistical expertise.

### Study subjects or cohort overlap

A recent publication has investigated ultrasound characteristics of ipsilateral axillary lymph nodes after two doses of four different COVID-19 (Igual-Rouilleault AC, Soriano I, Quan PL, Fernández-Montero A, Elizalde A, Pina L (2022) Axillary Lymph Node Imaging in mRNA, vector-based and mix-and-match COVID-19 vaccine recipients: Ultrasound features. *Eur Radiol*. https://doi.org/10.1007/s00330-022-08846-9). The 156 patients included in the cohort of this publication have also been included in this new research representing approximately 63% of the sample. However, in this project, our objective was to assess the association between ultrasound cortical thickening in reactive post-vaccination lymphadenopathy and the elicited humoral response and to evaluate the performance of cortical thickness as a predictor of vaccine *effectiveness in patients with and without previous history of* COVID-19 *infection.*

### Methodology

*•* prospective

*•* observational study

*•* performed at one institution
